# Guideline-based quality indicators—a systematic comparison of German and international clinical practice guidelines: protocol for a systematic review

**DOI:** 10.1186/s13643-017-0669-2

**Published:** 2018-01-12

**Authors:** Monika Becker, Jessica Breuing, Monika Nothacker, Stefanie Deckert, Mirco Steudtner, Jochen Schmitt, Edmund Neugebauer, Dawid Pieper

**Affiliations:** 1 Institute for Research in Operative Medicine (IFOM), Department for Evidence Based Health Service Research, Faculty of Health, Department of Medicine, Witten/Herdecke University, Ostmerheimer Str. 200, Building 38, 51109 Cologne, Germany; 2 AWMF-Institute for Medical Knowledge Management c/o Philipps-University Marburg, Karl-von-Frisch-Straße 1, 35043 Marburg, Germany; 3 Center for Evidence-based Healthcare, University Hospital and Medical Faculty Carl Gustav Carus, TU Dresden, Dresden, Germany; 4 Brandenburg Medical School – Theodor Fontane, Fehrbelliner Str.38, 16816 Neuruppin, Germany

**Keywords:** Guidelines, Quality indicator, Performance measures, Systematic review

## Abstract

**Background:**

Quality indicators (QIs) are used in assessing the quality of healthcare. Evidence-based clinical practice guidelines (CPGs) are relevant sources for generating QIs. In this context, QIs are important tools to assess the implementation of guideline recommendations. However, the methodological approaches to guideline-based QI development vary considerably.

In Germany, the guideline classification scheme of the AWMF (German Association of the Scientific Medical Societies) differentiates between S1-, S2k-, S2e-, and S3-CPGs depending on the methodological approach. Thus, S3-CPGs are consensus- and evidence-based CPGs and have the highest methodological standard in Germany. An analysis of the status quo of reported QIs in S3-CPGs found 35 current S3-CPGs, which report 372 different QIs.

Currently, there is no gold standard for the development of guideline-based QIs. To our knowledge, no studies have investigated to what extent guideline-based QIs from different CPGs that are related to the same topic are consistent. The objective of this study is to compare guideline-based QIs and their underlying methodological approaches of German S3-CPGs with those of topic-related international CPGs.

**Methods:**

Based on the previous identified German S3-CPGs (*n* = 35), which report quality indicators, we will conduct systematic searches in the guidelines databases of G-I-N (Guidelines International Network) and NGC (National Guideline Clearinghouse) to identify international CPGs matching the topics of the S3-CPGs. If necessary, we will search additionally the websites of the particular CPG providers for separate documents with regard to QIs. We will include evidence-based CPGs which report QIs. Reported QIs as well as methods of development and the rationale for QIs will be extracted and compared with those of the S3-CPGs.

**Discussion:**

This study will be part of the project “Systematic analysis of the translation of guideline recommendations into quality indicators and development of an evidence- and consensus-based standard,” supported by the German Research Association (DFG). The results of this analysis will feed into a subsequent qualitative study, which will consist of structured interviews with developers of international CPGs. Further, the results will be considered in a consensus study on standards of the translation of guideline recommendations into quality indicators in Germany.

**Electronic supplementary material:**

The online version of this article (10.1186/s13643-017-0669-2) contains supplementary material, which is available to authorized users.

## Background

Quality measurement and improvement play an important role in healthcare. For this purpose, quality indicators (QIs) can be used. There is no clear-cut definition of a QI. According to Lawrence and Frede, a QI is a “measurable element of practice performance for which there is evidence or consensus that it can be used to assess the quality, and hence change in the quality, of care provided” [1]. The Joint Commission on Accreditation of Healthcare Organizations (JCAHO) defines QIs as “[…] quantitative measures that can be used to monitor and evaluate the quality of important governance, management, clinical, and support functions that affect patient outcomes” [2]. To be deemed as trustworthy and useful, QIs have to satisfy different criteria, such as relevance, validity, reliability, feasibility, and target group orientation [3–6]. To meet the high methodological requirements on QIs, they should be based where possible on scientific evidence and developed in a systematic and transparent way [7, 8].

As evidence-based clinical practice guidelines (CPGs) are designed to reflect current best practice, they are relevant sources for generating QIs [7, 9]. The term “guideline-based QIs” indicates in particular QIs that are either generated from already available CPGs or coupled with the process of CPG development [10]. Besides assessing the quality of healthcare, these are important tools to assess the implementation of guideline recommendations [11–13]. However, the methodological approaches to guideline-based QI development vary considerably [10].

In Germany, the AWMF (German Association of the Scientific Medical Societies) provides the methodological framework for the development of CPGs by the scientific medical societies. The guideline classification scheme of the AWMF differentiates between S1-, S2k-, S2e-, and S3-CPGs depending on the methodological approach [14]. Thus, S1-CPGs are based on an informal consensus building. In S2k-CPGs, a formal consensus method is applied in a representative panel, and S2e-CPGs include a systematic approach for literature searching as well as selection and appraisal of evidence. S3-CPGs comprise both the requirements for S2k-CPGs and those for S2e-CPGs and thus have the highest methodological standard in Germany. An analysis of the status quo of reported QIs in S3-CPGs from 2013 found 34 S3-CPGs, which report 394 different QIs (including measures of quality which are labeled such as “quality criteria” or “quality measure”) [15]. For example, the S3-CPG “Diagnostics, treatment and follow-up care of malignant ovarial tumors” comprises 12 QIs, one of them regarding counseling by a social service (numerator: number of patients with counseling by a social service; denominator: all patients with an initial diagnosis of ovarian cancer and treatment in a clinical institution) [16]. In the S3-CPG “Long-Term Opioid-Use in Non-Cancer Pain,” three QIs are stated, such as the QI “number of patients with somatoform pain disorder who are treated with an opioid” [17]. An update (search up to 2016) of this analysis (not yet published) found 35 current S3-CPGs, which report 372 different QIs. Four S3-CPGs were developed by the National Program for Disease Management Guidelines (DMG), 15 by the German Guideline Program in Oncology (GGPO), and 16 by various medical societies. Particularly, the CPGs of the DMG and GGPO have a broad scope and cover various areas of medical care. For these CPGs, the development of guideline-based QIs is obligatory [11–13].

Although a working group of the Guidelines International Network (G-I-N) recently proposed a set of reporting standards for guideline-based performance measures [18], there is currently no gold standard for the development of guideline-based QIs [10, 19]. To our knowledge, no studies have investigated to what extent guideline-based QIs from different CPGs are consistent. Our hypothesis is that QIs from S3-CPGs are in many cases not corresponding with QIs of topic-related international CPGs.

The objective of this study is to compare guideline-based QIs and their underlying methodological approaches of the 35 previously identified German S3-CPGs with those of topic-related international CPGs.

## Methods

Our study is not registered with PROSPERO as we will not report health-related outcomes.

### Eligibility criteria

CPGs will be included in this study when they meet the following criteria:

1. QIs are reported
2. The CPG is an evidence-based CPG
3. The topic and recommendations have to be comparable with those of at least one of the 35 previous identified S3-CPGs (see Additional file 1)
4. Country of CPG development belongs to WHO-Stratum A [20]
5. Date of publication: between 2012 and 2017
6. Published in German, English, French, Spanish, Dutch, Norwegian, or Swedish
7. Current full-text version is available at no charge
8. The validity date of the CPG, indicated by the CPG developer, is not exceeded

If QIs are solely reported in a separate document, which is not a supplement to the CPG (e.g., evidence or methodological report), they have to be explicitly linked with the particular CPG. Otherwise, we will assume that these QIs are not guideline-based, and we will exclude the guideline. An example for such a separate document that contains guideline-based QIs is a document from the website of the National Institute for Health and Care Excellence (NICE): “NICE menu of general practice and clinical commissioning group indicators” [21]. The mentioned NICE-QIs usually are linked with particular CPGs (e.g., NICE guideline NG17). Evidence-based CPGs are defined in this analysis as guidelines whose recommendations are as follows:

- Based on a systematic literature search
- Clearly identifiable and with an assigned grade of recommendation (GoR) and/or a level of evidence (LoE)
- Explicitly or implicitly linked to the references of the underlying evidence

### Information sources and search strategy

Based on the previously identified S3-CPGs which report QIs, we will conduct systematic searches in the guidelines databases of G-I-N and NGC (National Guideline Clearinghouse) to identify international CPGs matching the topics of the S3-CPGs. The search strategies will include suitable keywords relating to the clinical topics and as appropriate truncations as well as Boolean operators. In cases where we cannot identify topical eligible guidelines, we will screen the websites of CPG providers additionally, whereby the searches will be tailored to the structure and capabilities of the websites. Furthermore, we will crosscheck the reference lists of the S3-CPGs and the international CPGs eligible for inclusion in the analysis.

In cases where topical eligible CPGs comprise neither QIs nor links to QIs, we will search the websites of the particular CPG providers for separate documents with regard to QIs that are explicitly linked with the particular CPG.

### Data management and selection process

One reviewer will screen the titles of records, and the full texts of those deemed eligible for inclusion will be retrieved. In the next step, the screening of full texts will be conducted by one reviewer and checked by another. The reasons for exclusion will be documented, and any disagreements will be resolved through discussion and consensus.

The records will be uploaded and managed using Microsoft Excel.

In cases where no eligible CPG matching the topic of a S3-CPG can be found, we will exclude the particular CPG from analysis.

### Data collection process and data items

A standardized extraction form will be developed based on the data extraction items used in a preliminary project [15] and pilot-tested. The following information will be collected:

- Information on QI-development group (number of members and positions, such as methodologists, clinicians, patient representatives)
- Labeling of the measure of quality, e.g., QI, quality criteria, performance measure
- Categorization of QI in structure, process, outcome indicator according to the definition of Donabedian [22] (in case of missing assignment by the guideline authors an own assignment will be made)
- Underlying recommendations, if the QIs are based explicitly or implicitly on those
- Reported rationale for the QI
- Reported measurement properties of QI, e.g., reliability and validity [23]
- Reported intended purpose of QI, e.g., quality reporting, quality management systems, evaluation of CPGs
- Reported quality objectives
- Methods of QI-development, e.g., searches for existing QIs, consensus methods, assessment-tools

The extractions will be conducted by one reviewer and checked by another, any disagreements will be resolved through discussion and consensus.

### Quality appraisal

As a high methodological quality of CPGs is asked to be a source of high quality and trustworthy guideline-based QIs [10, 18], the methodological quality of all included CPGs will be appraised using the domain “Methodological Rigor of Development” of the German Instrument for Methodological Guideline Appraisal (DELBI) [24]. Seven items will be rated on a 4-point scale (whereby one = “strongly disagree,” two = “disagree,” three = “agree,” and four = “strongly agree”):

- Systematic methods were used to search for evidence
- The criteria for selecting the evidence are clearly described
- The methods used for formulating the recommendations are clearly described
- Health benefits, side effects, and risks have been considered in formulating the recommendations
- There is an explicit link between the recommendations and the supporting evidence
- The guideline has been externally reviewed by experts prior to its publication
- A procedure for updating the guideline is provided

Two reviewers will perform the quality assessment independently. In case of two or more points of difference in the appraisal of the two reviewers, disagreement will be resolved through discussion and consensus. A domain score will be calculated by summing up the scores of the individual items and by standardizing the total as the percentage of the maximum possible score for the domain (4 (strongly agree) × 7 (items) × 2 (appraisers)) [24].

Reviewers who have been involved in the development of the included CPGs will not participate in their quality assessment.

### Data synthesis

Data synthesis will contain a descriptive analysis and a tabular comparison of the QIs of the included CPGs and those of the S3-CPGs for each clinical topic and when applicable for each underlying recommendation. We will collect the number of CPGs that give information to the QI-development group, the methods of QI-development, as well as the rationale and intended purpose of QI. On the basis of reported QIs, we will collect the number of QI for which quality objectives and measurement properties are reported as well as the number of QI that are explicitly or implicitly based on guideline recommendations.

For each matched pair of CPGs, we will compare the suggested QIs and assess if the QIs agree, disagree, or if they are not comparable. We will assign QIs on the same topic either to the category “not different/slightly different” or “different.” QIs that are not comparable will be extracted under the category “QI only defined in the international respectively the S3-CPG. For each category, we will collect the number of QIs respectively QI-pairs. Furthermore, the methods for QI-development will be summarized narratively.

## Discussion

This study will be part of the project “Systematic analysis of the translation of guideline recommendations into quality indicators and development of an evidence- and consensus-based standard,” supported by the German Research Association (DFG). It will be the second systematic analysis in the overall project. The results of this analysis will feed into a subsequent qualitative study which will consist of structured interviews with developers, methodologists, and users of international guidelines. Both studies intend to deliver additional information to existing research on methods for the development of guideline-based QIs [10, 18]. For the analysis of possible differences between QIs from different CPGs, we will consider existing guidelines or rather QI development manuals of the respective guideline organization.

**Fig. 1:**
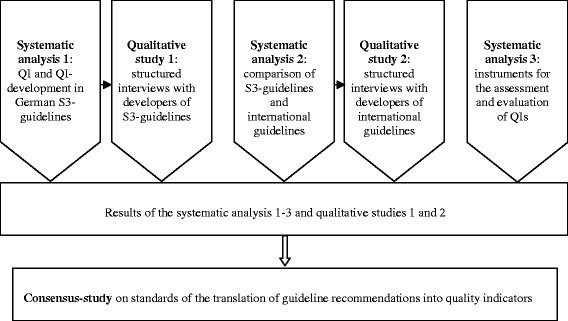
Overview of the overall project. QI = quality indicator

An overview of the overall project is shown in Fig. 1.

### Presenting and reporting the results

This protocol adheres to the “Preferred Reporting Items for Systematic Review and Meta-Analysis-Protocols (PRISMA-P)” [25]. As PRISMA-P aims to guide the development of protocols for systematic reviews evaluating therapeutic efficacy, we deviated from the original checklist by omitting items (e.g., outcomes and prioritization) due to the methodological focus of our planned systematic review (see Additional file 2 for the completed PRISMA-P checklist).

The results of our study will be considered in the last phases of the overall project, namely a consensus-study on standards of the translation of guideline recommendations into quality indicators.

## Additional files

Additional file 1: S3-CPGs which report QIs. (DOCX 25 kb)

Additional file 2: PRISMA-P 2015 Checklist. (DOCX 35 kb)

## Abbreviations

AWMF: German Association of the Scientific Medical Societies
CPG: Clinical practice guideline
DELBI: German Instrument for Methodological Guideline Appraisal
G-I-N: Guidelines International Network
NGC: National Guideline Clearinghouse
PRISMA-P: Preferred Reporting Items for Systematic Review and Meta-Analysis-Protocols
QI: Quality indicator
