# Supplementary material for: Guideline-based quality indicators—a systematic comparison of German and international clinical practice guidelines: protocol for a systematic review
Source: Syst Rev. 2018 Jan 12;7:5. doi: 10.1186/s13643-017-0669-2 (PMC5767020; doi:10.1186/s13643-017-0669-2)
Supplement: Supplementary file 1 — S3-CPGs which report QIs. (DOCX 25 kb) [file 13643_2017_669_MOESM1_ESM.docx]

**Additional file 1: S3-CPGs which report QIs** (available under <http://www.awmf.org/leitlinien/>)

1. Analgesie, Sedierung und Delirmanagement in der Intensivmedizin (Management of delirium, analgesia and sedation in intensiv care medicine); register number: 001 – 012
2. Alkoholbezogene Störungen: Screening, Diagnose und Behandlung (Screening, diagnostics and therapy of alcohol related disorders*); register number: 076-001
3. Pneumonie, ambulant erworben, Behandlung und Prävention von erwachsenen Patienten (Management of adult community-acquired pneumonia and prevention); register number: 020 – 020
4. Diabetes und Schwangerschaft (Diabetes and pregnancy); register number: 057 – 023
5. Bipolare Störungen (bipolar disorder*); register number: 038 – 019
6. Karpaltunnelsyndrom; Diagnostik und Therapie (Clinical diagnostics and treatment of Carpal tunnel syndrome*); register number: 005 – 003
7. Epidurale Rückemarksstimulation zur Therapie chronischer Schmerzen (epidural spinal cord stimulation as treatment for chronic pain*), register number: 008 – 023
8. Diagnostik, Therapie und Nachsorge (Diagnostics, treatment and follow-up care of gestational diabetes mellitus*); register number: 057 – 008
9. Indikation und Methodik der Hysterektomie bei benignen Erkrankungen (Indicationand technics of hysterectomy for benigne diseases*); register number: 015 – 070
10. Opioide, Langzeitanwendung zur Behandlung bei nicht tumorbedingten Schmerzen (Long-Term Opioid-Use in Non-Cancer Pain); register number: 145 – 003
11. Müdigkeit (tiredness*); register number: 053 – 002
12. Adipositas - Prävention und Therapie (Prevention and treatment of adiposity*); register number: 050-001
13. S3-Leitlinie Prophylaxe der venösen Thromboembolie (VTE) (Prophylaxis of venous thromboembolism*); register number: 003 – 001
14. Strategien zur Sicherung rationaler Antibiotika-Anwendung im Krankenhaus (Strategies for ensuring rational antibiotic use in hospitals*); register number: 092- 001
15. Vermeidung von perioperativer Hypothermie (Avoidance of peroperative hypothermia*); register number: 001 – 018
16. Versorgung peripherer Nervenverletzungen (Management of peripheral nerve injuries*); register number: 005 – 010
17. Nationale VersorgungsLeitlinie Nicht-spezifischer Kreuzschmerz (low-back pain*); register number: nvl – 007
18. Nationale VersorgungsLeitlinie Typ-2-Diabetes: Schulung (Type 2 diabetes: education*); register number: nvl - 001f
19. Nationale VersorgungsLeitlinie Nierenerkrankungen bei Diabetes im Erwachsenenalter (Kidney disease in diabetes in adulthood*); register number: nvl - 001d
20. Nationale VersorgungsLeitlinie Neuropathie bei Diabetes im Erwachsenenalter (Neuopathy in diabetes in adulthood*); register number: nvl - 001e
21. Magenkarzinom - Diagnostik und Therapie der Adenokarzinome des Magens und ösophagogastralen Übergangs (Diagnostics and treatment of adenocarcinomas of the stomach and oesophagogastric junction); register number: 032 - 009OL
22. Mundhöhlenkarzinom; Diagnostik und Therapie (Diagnostics and treatment of oral cancer*); register number: 007-100OL
23. Mammakarzinom der Frau; Diagnostik, Therapie und Nachsorge (Diagnostics, treatment and follow-up care of breast cancer*); register number: 032 - 045OL
24. Hepatozelluläres Karzinom; Diagnostik und Therapie (Diagnostics and treatement of heaptocellular carcinoma); register number: 032/053OL
25. Hodgkin Lymphom; Diagnostik, Therapie und Nachsorge von erwachsenen Patienten (Diagnostics, treatment and follow-up care of adult Hodkin Lymphoma*); register number: 018/029OL
26. Maligne Ovarialtumore; Diagnostik, Therapie und Nachsorge (Diagnostics, treatment and follow-up care of malignant ovarial tumors*); register number: 032 - 035OL
27. Exokrines Pankreaskarzinom (exocrine pancreatic carcinoma*); register number: 032/010OL
28. Diagnostik, Therapie und Nachsorge der Patientin mit Zervixkarzinom (Diagnostics, treatment and follow-up care of the patient with cervical carcinoma*); register number: 032 - 033OL
29. Interdisziplinäre Leitlinie der Qualität S3 zur Früherkennung, Diagnose und Therapie der verschiedenen Stadien des Prostatakarzinoms (Early diagnosis, Diagnostics and treatment of various states of prostate cancer*); register number: 043 - 022OL
30. Kolorektales Karzinom (Guideline for Colorectal Cancer); register number: 021 - 007OL
31. Psychoonkologische Diagnostik, Beratung und Behandlung von erwachsenen Krebspatienten (psychooncologic diagnostic, counselling and treatment of adult cancer patients*); register number: 032/051OL
32. Plattenepithelkarzinome und Adenokarzinome des Ösophagus; Diagnostik und Therapie (Diagnostics and treatment of squamous cell carcinomas and adenocarcinomas of the oesophagus*); register number: 021/023OL
33. Nierenzellkarzinom; Diagnostik, Therapie und Nachsorge (Diagnostics, treatment and follow-up care of the renal cell carcinoma*), register number: 043 - 017OL
34. Palliativmedizin für Patienten mit einer nicht heilbaren Krebserkrankung (palliative care for patients with incurable cancer); register number: 128 - 001OL
35. Malignes Melanom; Diagnostik, Therapie und Nachsorge (Diagnostics, treatment and follow-up care of the melanoma*); register number: 032-024OL

* translated freely
